# *In silico* Approach for Unveiling the Glycoside Hydrolase Activities in *Faecalibacterium prausnitzii* Through a Systematic and Integrative Large-Scale Analysis

**DOI:** 10.3389/fmicb.2019.00517

**Published:** 2019-04-04

**Authors:** Guillermo Blanco, Borja Sánchez, Florentino Fdez-Riverola, Abelardo Margolles, Anália Lourenço

**Affiliations:** ^1^ESEI – Department of Computer Science, University of Vigo, Ourense, Spain; ^2^Department of Microbiology and Biochemistry of Dairy Products, Superior Council of Scientific Investigations, Institute of Dairy Products of Asturias, Villaviciosa, Spain; ^3^SING Research Group, Galicia Sur Health Research Institute (IIS Galicia Sur), SERGAS-UVIGO, Vigo, Spain; ^4^Centre for Biomedical Research, University of Vigo, Vigo, Spain; ^5^Centre of Biological Engineering, University of Minho, Braga, Portugal

**Keywords:** fermentable sugars, glycoside hydrolases, bioactivity, *Faecalibacterium prausnitzii*, computational screening

## Abstract

This work presents a novel *in silico* approach to the prediction and characterization of the glycolytic capacities of the beneficial intestinal bacterium *Faecalibacterium prausnitzii*. Available *F. prausnitzii* genomes were explored taking the glycolytic capacities of *F. prausnitzii* SL3/3 and *F. prausnitzii* L2-6 as reference. The comparison of the generated glycolytic profiles offered insights into the particular capabilities of *F. prausnitzii* SL3/3 and *F. prausnitzii* L2-6 as well as the potential of the rest of strains. Glycoside hydrolases were mostly detected in the pathways responsible for the starch and sucrose metabolism and the biosynthesis of secondary metabolites, but this analysis also identified some other potentially interesting, but still uncharacterized activities, such as several hexosyltransferases and some hydrolases. Gene neighborhood maps offered additional understanding of the genes coding for relevant glycoside hydrolases. Although information about the carbohydrate preferences of *F. prausnitzii* is scarce, the *in silico* metabolic predictions were consistent with previous knowledge about the impact of fermentable sugars on the growth promotion and metabolism of *F. prausnitzii*. So, while the predictions still need to be validated using culturing methods, the approach holds the potential to be reproduced and scaled to accommodate the analysis of other strains (or even families and genus) as well as other metabolic activities. This will allow the exploration of novel methodologies to design or obtain targeted probiotics for *F. prausnitzii* and other strains of interest.

## Introduction

The high complexity of the human gut microbiota and the lack of know-how on all the individual players is a key challenge in gut microbiome research. Attempts have been made to define the microorganisms that constitute a healthy human gut microbiota but the scientific community has not yet reached a consensus to define the human beneficial intestinal microbial fingerprint. However, a number of observational studies have repeatedly shown a correlation between some bacterial populations and different physiological states, including those having an influence on human health. Specifically, some groups of strict anaerobes, such as *Faecalibacterium* *prausnitzii*, are found to be underrepresented in physiological conditions in which inflammation and oxidative stress are present (Joossens et al., 2011; Morgan et al., 2012; Gevers et al., 2014). Thus, during the last decade, *F.* *prausnitzii* research experienced a boosting, and this microorganism emerged as one of the most promising next-generation probiotics (O’Toole et al., 2017).

The species *Fusobacterium prausnitzii* was reclassified in 2002 by Stewart and co-workers, who proposed the new species *F. prausnitzii* (Stewart et al., 2002). *F. prausnitzii* is highly prevalent in the large intestine of humans and represents a significant proportion of the fecal anaerobes that produce major quantities of butyrate (Sokol et al., 2008; Arumugam et al., 2011). It is part of the autochthonous mucosal bacteria inhabiting the colon and its population has been found to be decreased in several diseases, particularly in those with mucosal inflammation, such as the inflammatory bowel disease or the type 2 diabetes (Ahmed et al., 2007; Sokol et al., 2008; Machiels et al., 2014; Tilg and Moschen, 2014; Rossi et al., 2016). In fact, it was demonstrated that *F. prausnitzii* has anti-inflammatory activity, likely due to its capacity to induce the secretion of the anti-inflammatory cytokine IL-10 in human immune cells and thus modulate T-cell responses (Rossi et al., 2016), as well as to produce microbial anti-inflammatory proteins able to inhibit the NF-κB pathway in intestinal epithelial cells (Quévrain et al., 2016), or extracellular polysaccharides able to attenuate clinical parameters of colitis (Rossi et al., 2015).

Microbiota studies using massive sequencing methodologies, either metataxonomics or metagenomics analyses, point to a high abundance of *Faecalibacterium* in the human gut (Arumugam et al., 2011). However, since *F. prausnitzii* is very sensitive to low oxygen concentrations and possesses a low technological robustness for industrial purposes, it is difficult to isolate from fecal samples and to grow using traditional cultivation methods. Also, although different phylogroups can be distinguished within the species (Benevides et al., 2017), the phenotypic characterization of strains has been restricted to a few isolates.

Metabolic maps have been generated using as the strain A2-165 (DSM17677T) as reference (El-Semman et al., 2014; Rowland et al., 2018), but the *F. prausnitzii* pangenome has never been explored to describe the metabolic capabilities of the genus and be able to develop novel microbial cultivation strategies. Therefore, the aim of the present work was to investigate the genome-based metabolic potential of *F. prausnitzii*, based on a deep prediction of the glycolytic capacities of this bacterium. The *F. prausnitzii* genomes currently deposited in the NCBI Genome Database were explored, taking the metabolisms of *F. prausnitzii* SL3/3 and *F. prausnitzii* L2-6 and data on all known glycoside hydrolases as references. To the best of our knowledge, this is the first time that a high throughput bioinformatics approach is carried out to predict the glycolytic capacities of *F. prausnitzii*.

## Materials and Methods

**FIGURE 1:**
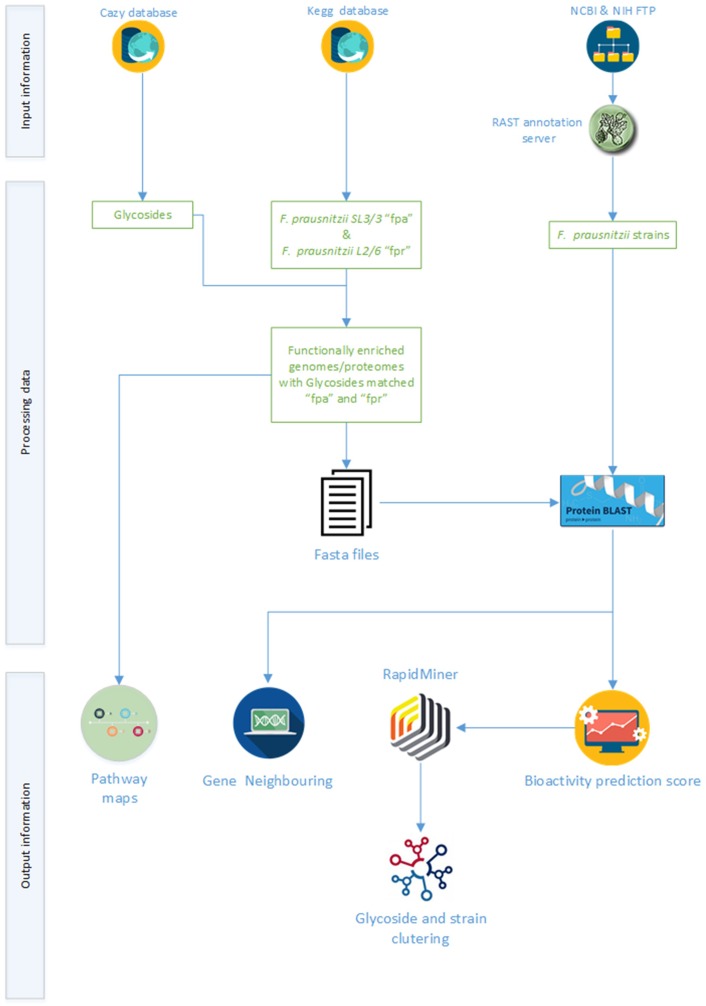
Schematic of the computational workflow supporting the analysis of the glycolytic potential of available *F. prausnitzii* genomes.

The primary aim of the computational workflow developed in this work was to support the investigation of glycoside hydrolase activities in *F. prausnitzii*. Complimentarily, programmatic access to public data sources and the use of broad scope genomic and metabolic tools were sought as means to make this study completely reproducible as well as be able to apply this workflow to similar studies in the future. The proposed workflow (Figure 1) encompasses three main steps, i.e., data retrieval, data integration and data analysis. The primary data sources were the carbohydrate-active enzymes database (CAZy), the kyoto encyclopedia of genes and genomes (KEGG), and the Genome database of National center for biotechnology information (NCBI). Data integration was based on the matching of the glycoside hydrolase sequences against the sequences of the proteins encoded in the genomes of *F. prausnitzii* strains. As a rule of thumb, a threshold of 80% was used as baseline to identify most prominent activities in the different strains. Protein homology data were used to segregate clusters of glycoside hydrolases and strains for further pattern analysis (notably, based on the obtained identity percentage). Moreover, gene neighborhood maps were generated for the genes coding for some relevant glycoside hydrolases. Experts curated the results obtained throughout the execution of the workflow towards presenting a comprehensive portrait of current knowledge on glycoside hydrolases activities in *F. prausnitzii* as well as ensuring the validity of the overall process of analysis. The next sections describe the main steps in the workflow, including processing statistics, methods, and Supplementary Material produced during the analysis.

### Glycoside Hydrolases and Reference Strains

Data on the glycoside hydrolase families were retrieved from the CAZy (Cantarel et al., 2009). From the families present in the CAZy database, only those that have associated fully specified Enzyme Commission numbers (EC number) (Tipton, 1994) were considered (i.e., enzyme codes including four numerical classes separated by periods).

Data on the metabolism of *F. prausnitzii* strains were retrieved from the KEGG (Kanehisa et al., 2017) using its programmatic API. Two strains of *F. prausnitzii* were used as reference in the present study, i.e., *F. prausnitzii* SL3/3 (identified hereinafter as “fpa”) and *F. prausnitzii* L2-6 (identified hereinafter as “fpr”). Protein and gene information was retrieved for all the previously selected glycoside hydrolases, including definition (i.e., reference name in GenBank database) (Benson et al., 2018), pathway, amino acid sequence and nucleotide sequence. The KEGG Mapper^1^ was applied to generate the pathway maps for both strains. The lists of amino acid sequences of glycoside hydrolases found in “fpa” and “fpr” were gathered in two fasta files (see Supplementary Table S1).

Publicly available genomes of *F. prausnitzii* strains were retrieved from the Genome database of the NCBI via FTP (Agarwala et al., 2018). From a total of 27 strains, 15 strains have been released in 2017 and the protein annotations are not yet available. Therefore, these genomes required further annotation. The rapid annotation using subsystem technology (RAST) platform was used for this purpose (Overbeek et al., 2014).

### Homology Analysis

The basic local alignment search tool (BLAST), most notably the Blastp 2.6.0 tool, supported the search of glycoside hydrolase sequences in the compiled *F. prausnitzii* genomes (Altschul et al., 1997). A Matlab script was coded to generate the results, generally referred to as “fpa” (i.e., *F. prausnitzii* SL3-3 as reference) and “fpr” (i.e., *F. prausnitzii* L2-6 as reference), and obtain the homology data for each of the strains. Specifically, data on the percentage identity, the alignment length, the number of mismatches, gap opens, the start and end of alignment in query/subject, the expected value, and the bit score.

A heatmap-alike representation of the best homology values with associated hierarchical clustering (Euclidean distance and average linkage) was further implemented to support a more intuitive inspection of the results. Values of homology below 50% were suspected to represent non-orthologs enzymes. Conversely, homology values close to 1 were investigated as likely pointing to orthologs enzymes. Supplementary Table S2 details all these data.

### Clustering *F. prausnitzii* Strains

The k-means partitioning clustering method was applied to further analysis of the homology results, most notably to identify groups of strains showing similar traits (Neyman, 1967). This analysis included the clustering of glycoside hydrolases activities according to their prevalence in the analyzed strains as well as the clustering of strains according to the predicted glycoside hydrolases activities.

Distance similarity was based on the Euclidean metric. Several values of *k* were analyzed for each scenario and the best number of clusters was determined based on the rule of thumb:

(1) k∼n2

where *n* being the number of instances to cluster, and the Davies-Bouldin index, which evaluates intra-cluster similarity (i.e., the members of a cluster should present rather similar traits), and inter-cluster differences (i.e., clusters should be reasonably different) (Davies and Bouldin, 1979). Specifically, the number of clusters (*k*) was initially set to 4 and then fine-tuned using the Davies-Bouldin index (in general, the lowest the better). See Supplementary Table S3 for details on this analysis.

Rapid Miner 8.2 software platform was used to conduct the clustering experiments (Hofmann and Klinkenberg, 2013). The graphical representation of the results was produced using a Python script, based on the graphical libraries NumPy (van der Walt et al., 2011) and Matplotlib (Hunter, 2007).

### Gene Neighboring

The SEED genome annotation viewer^2^ was used to investigate the gene neighborhoods related to glycoside hydrolase activities. SEED had publicly available annotations for 5 of 27 strains under analysis, i.e., the strains A2-165, M21/2, SL3/3, L2-6, and KLE1255. In-house experts, via the RAST server^3^, annotated the other 22 genomes (managed as a private annotation project in SEED). Next, the SEED Viewer tool^4^ was used to create a graphical representation of the gene neighborhood for the genes encoding glycoside hydrolases in *F. prausnitzii* or related strains.

Supplementary Table S4 lists the SEED URLs for public inspection. While it is not possible to supply public links for the genomes annotated privately, this document explains how to reproduce the analysis at its full extent.

## Results

### Metabolic Portrait of *F. prausnitzii*

Currently, KEGG database describes the metabolism of *F. prausnitzii* SL3/3 (identified as “fpa,” and documenting 2820 genes and 2756 proteins) and *F. prausnitzii* L2-6 (identified as “fpr,” and documenting 2816 genes and 2746 proteins). In particular, KEGG describes 90 metabolic pathways in these organisms. Glycoside hydrolases are annotated in 17 pathways of *F. prausnitzii* SL3/3 and 14 pathways of *F. prausnitzii* L2-6, respectively (Table 1). For the most part, glycoside hydrolases are located in the pathways responsible for the starch and sucrose metabolism and the biosynthesis of secondary metabolites.

**Table 1:** Pathways of the metabolisms of *F. prausnitzii* SL3/3 and *F. prausnitzii* L2-6 containing glycoside hydrolases.

	Number of glycoside hydrolase	
	
Pathways	Fpa	Fpr
Starch and sucrose metabolism	17	15
Biosynthesis of secondary metabolites	15	13
Galactose metabolism	7	8
Purine metabolism	6	6
Pyrimidine metabolism	6	6
Sphingolipid metabolism	6	0
Other glycan degradation	4	4
Pentose and glucuronate interconversions	2	2
Cyanoamino acid metabolism	3	2
Pentose and glucuronate interconversions	2	0
Glycerolipid metabolism	2	0
Nicotinate and nicotinamide metabolism	2	2
Porphyrin and chlorophyll metabolism	2	2
Uridine monophosphate biosynthesis, glutamine (+PRPP) ⇒ UMP	0	2
Glycolysis/Gluconeogenesis	3	1
Alanine, aspartate, and glutamate metabolism	1	1
Biosynthesis of antibiotics	1	1
Cobalamin biosynthesis, cobinamide = >cobalamin	1	0


**FIGURE 2:**
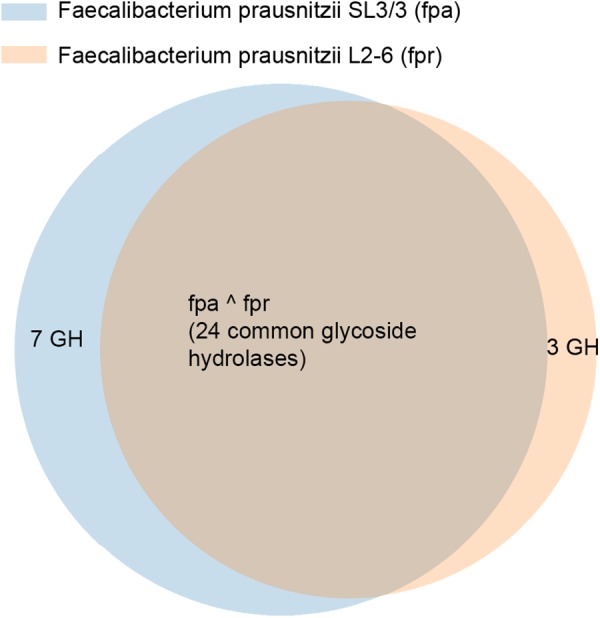
Relation of distribution of glycoside hydrolases in *F. prausnitzii* SL3/3 and *F. prausnitzii* L2-6.

Figure 2 illustrates the gene orthology and the glycolytic activities in the two strains, as described in KEGG. *F. prausnitzii* SL/3 has 44 glycoside coding genes and *F. prausnitzii* L2-6 has 37 of such genes. 27 of these genes are orthologs, whilst 8 of the genes are specific of *F. prausnitzii* SL/3 and 3 are specific of *F. prausnitzii* L2-6. As a result, the two strains have in common 24 glycoside hydrolases. In terms of specific activity in *F. prausnitzii* SL3/3, this can be pin pointed to the pathways related with sphingolipid metabolism, the pentose and glucuronate interconversions, the glycerolipid metabolism, and the cobalamin biosynthesis (i.e., the glycoside hydrolases 2.4.1.20, 2.4.1.281, 2.4.1.319, 2.4.1.320, 3.2.1.22, 3.2.1.40, and 3.2.1.122). Likewise, the glycoside hydrolases 2.4.1.4, 3.2.1.10, and 3.2.1.26 show activity only in the uridine monophosphate biosynthesis of *F. prausnitzii* L2-6.

**FIGURE 3:**
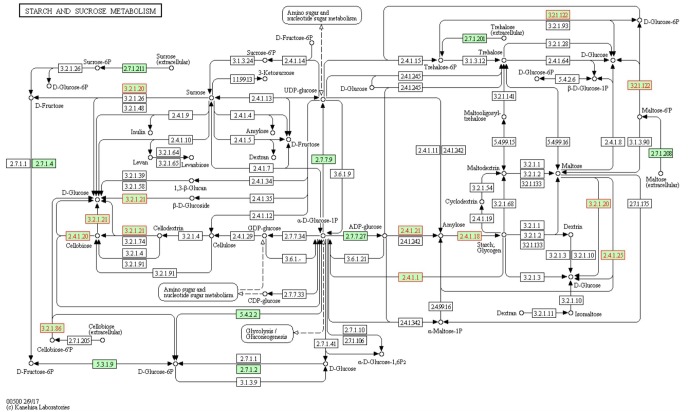
Metabolic map of the starch and sucrose metabolism in *F. prausnitzii* SL3/3. The EC numbers colored in green are organism-specific and red colored font highlights glycoside hydrolase activities

As an example, Figure 3 details the enzymatic activities found in the starch and sucrose metabolism of *F. prausnitzii* SL3/3. The program implemented in-house to generate the pathway maps using the KEGG Mapper depicts the enzymatic activities of interest as well as organism-specific activity. That is, organism-specific reactions are colored in green and glycoside hydrolases are colored in red. All objects have links to the corresponding KEGG entries.

### Homology Analysis

**FIGURE 4:**
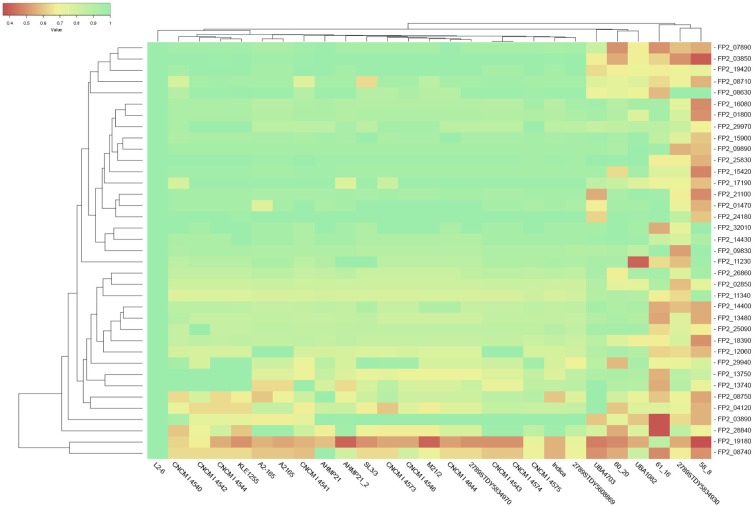
Extract of the heatmap representation of the homology results for *F. prausnitzii* SL3/3. The rows describe glycoside hydrolases and the columns depict the *F. prausnitzii* strains under analysis. The color hue of the cells represents the magnitude of the obtained homology score (red for 0% to green for 100%).

The results of the sequence similarity analysis, in particular the percentage identity data, were further analyzed. Figure 4 illustrates the results obtained having *F. prausnitzii* SL3/3 (fpa) as reference. Table 2 describes the glycoside hydrolase activities that have broader, acceptable, and lower probability of being present in the *F. prausnitzii* strains analyzed. A threshold of 50% of homology guided the analysis of gene orthology, i.e., the likelihood of the same gene being present across multiple strains.

**Table 2:** Glycoside hydrolases showing very high (>98%), acceptable (>75%), and low values of homology (<50%) having the *F. prausnitzii* SL3/3 strain as reference.

%	Glycoside hydrolase	Number strains	Enzyme	Pathway
>98	FPR_06900	16 strains	EC 3.1.1.29	Non-existent information
	FPR_27540	16 strains	EC 3.2.1.23	Galactose metabolism (fpa00052)
				Other glycan degradation (fpa00511)
				Sphingolipid metabolism (fpa00600)
	FPR_27530	15 strains	EC 2.4.1.211	Non-existent information
>75	FPR_13470	All 27 strains	EC 2.4.1.21	Starch and sucrose metabolism (fpa00500)
				Biosynthesis of secondary metabolites (fpa01110)
	FPR_05900	26 strains (except 60_20)	EC 2.4.1.20	Starch and sucrose metabolism (fpa00500)
	FPR_14930	26 strains	EC 3.2.1.86	Glycolysis/Gluconeogenesis (fpa00010)
		(except 61_16)		Starch and sucrose metabolism (fpa00500)
<50	FPR_17220	L26 58_8 60_20 61_16	EC 2.4.1.281	Non-existent information
	FPR_25170	A2165 CNCM I 4543 CNCM I 4574	EC 3.2.1.86	Glycolysis/Gluconeogenesis (fpa00010) Starch and sucrose metabolism (fpa00500)
	FPR_25480	58_8 60_20	EC 3.2.1.21	Cyanoamino acid metabolism (fpa00460)
				Starch and sucrose metabolism (fpa00500)
				Biosynthesis of secondary metabolites (fpa01110)


Three glycoside hydrolases showed a perfect homology score (98%) for almost all the strains namely: a beta-galactosidase/beta-glucuronidase (FPR_27540) that participates in the galactose and sphingolipid metabolisms (fpa00052 and fpa00600, respectively) as well as in glycan degradation (fpa00511); an uncharacterized peptidyl-tRNA hydrolase (FPR_06900); and, a conserved hypothetical 1,3-beta-galactosyl-N-acetylhexosamine phosphorylase (FPR_27530).

Various other glycoside hydrolases showed homology scores above 75% for (almost) all strains. Notably, a starch synthase (FPR_13470), a cellobiose phosphorylase (FPR_05900), and a 6-phospho-beta-glucosidase (FPR_14930) are highlighted as being potentially present in practically all the studied strains (Table 2). The starch and sucrose metabolism (fpa00500) is again in evidence, but these enzymes are also present pathways such as the glycolysis/gluconeogenesis (fpa00010) and the biosynthesis of secondary metabolites (fpa01110).

A predicted 4-O-beta-D-mannosyl-D-glucose phosphorylase (FPR_17220), a 6-phospho-beta-glucosidase (FPR_25170), and a beta-glucosidase (FPR_25480) were among those glycoside hydrolases that showed values of homology below 50%. FPR_17220 is a predicted glycoside hydrolase that was found in only 4 of the strains with a score of no more than 48%. FPR_25170 is a glycoside hydrolase located in the glycolysis/gluconeogenesis metabolism (fpa00010) and the starch and sucrose metabolism (fpa00500), and did not achieve any result above 45%. Finally, FPR_25480 is an aryl-beta-glucosidase, which is present in the starch and sucrose metabolism (fpa00500), the cyanoamino acid metabolism (fpa00460) and the biosynthesis of secondary metabolites (fpa01110). The prediction scores of these glycoside hydrolases were below 45%. Also noteworthy, the aryl-beta-glucosidase (FPR_25480) is the glycoside hydrolase predicted in fewer strains, i.e., 2 out of the 27 strains, even though homology scores are greater than 50%.

The strains CNCM I 4546 and M21/2 showed the most overall homology with respect to the reference strain (SL3/3). They show homology values above 75% for all glycoside hydrolases, with the exception of the 6-phospho-beta-glucosidase (i.e., FPR_25170) in M21/2. Conversely the strain 58_8 showed the lowest overall homology results, i.e., 32 out of the 44 glycoside hydrolases have homology results below 70%. Supplementary Table S2 details these data.

### Clustering of *F. prausnitzii* Strains

Previous works proposed possible divisions of the *Faecalibacterium* group based on phylogenetic analyses (Benevides et al., 2017; Fitzgerald et al., 2018). In this study, the clustering of the studied strains was more specialized, i.e., it was based on the homology results for the glycoside hydrolases. Therefore, here, the strains clustered together have similar homology profiles for the glycoside hydrolases (i.e., glycolytic traits) whereas the clusters represent different, potentially meaningful sets/combinations of glycolytic activities.

**FIGURE 5:**
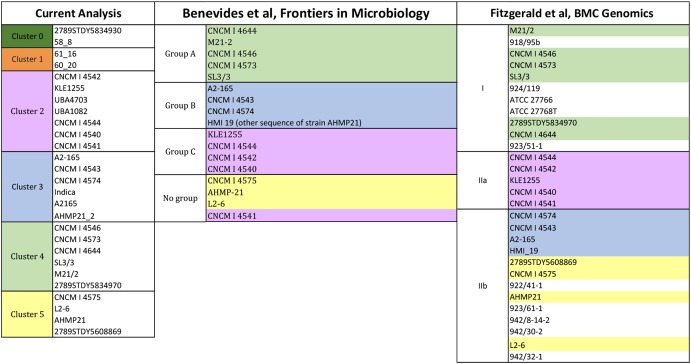
Clustering of strains activities having *F. prausnitzii* SL3-3 (fpa) as reference. The reference strains are located in cluster 4. Strain per cluster: cluster 0 (58_8, 2789STDY5834930), in cluster 1 (60_20 and 61_16), in cluster 2 (UBA4703, UBA1080, CNCM | 4544, KLE1255, CNCM | 4542, CNCM | 4540, and CNCM | 4541), in cluster 3 (A2-165, Indica, CNCM | 4574, A2165, CNCM | 4543, and AHMP21-2), in cluster 4 (M21/2, CNCM | 4573, 2789STDY5834970, SL3/3, CNCM | 4546, and CNCM | 4644), and in cluster 5 (CNCM | 4575, L2-6, AHMP21, and 2789STDY5608869).

Figure 5 describes the clustering of the *F. prausnitzii* strains based on the glycolytic traits as well as phylogeny. The strains present in the glycolytic study are also in the reference phylogenetic studies, with the exception of the strains 2789STDY5834930, 58_8 and 61_16. Color notation helps to compare the present homology-based clustering to two phylogenetic groupings. Overall, most of the groupings are in accordance to one another. The most noticeable difference is that the strain CNCM I 4541 is grouped apart from the rest of the strains showing similar glycolytic traits (i.e., cluster 4, light green colored) in both phylogenetic studies. This glycolytic group showed a high prevalence of glycosides participating in the glycolysis/gluconeogenesis as well as the metabolism of starch and sucrose.

The strains in the cluster 1 (orange colored) and the cluster 2 (pink colored) showed a high frequency of some glycosyltransferases and presence in the pyrimidine metabolism. The biggest difference between these clusters is that cluster 1 shows a low presence of some hexosyltransferases (i.e., FPR_17220 and FPR_17240) whereas cluster 2 is characterized by a low presence of a beta-glucosidase (the FPR_25480), which is present in the starch and sucrose metabolism as well as the cyanoamino acid metabolism.

The strains in the cluster 3 (blue colored) showed low scores for FPR_25170 and high scores for FPR_12120, both glycosides associated with the starch and sucrose metabolism. In turn, the strains of the cluster 4 (light green colored) and the cluster 5 (yellow colored) display overall high frequency profiles. These strains show high homology for enzymes associated to the pyrimidine metabolism. Differences lay in the high score of a 4-alpha-glucanotransferase (FPR_24180) in cluster 4, which participates in the starch and sucrose metabolism, whereas the key glycoside hydrolase in cluster 5 is a purine-nucleoside phosphorylase (FPR_06940), which is part of the metabolisms of purine and the metabolism of nicotinate and nicotinamide.

**FIGURE 6:**
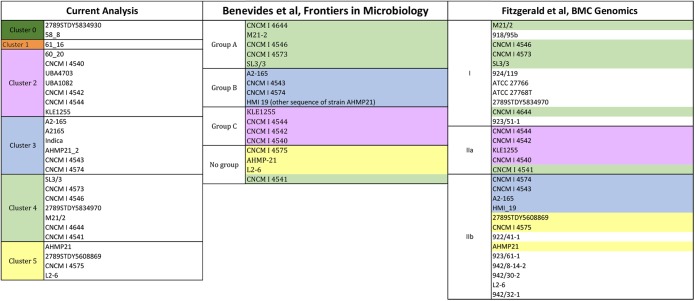
Clustering of strains having *F. prausnitzii* L2-6 (fpr) as reference. The reference strains is located in cluster 5. Strains per cluster: cluster 0 (58_8 and 2789STDY5834930), in cluster 1 (61_16), in cluster 2 (UBA1082, UBA4703, 60_20, CNCM | 4540, CNCM | 4542, CNCM | 4544, and KLE1255), in cluster 3 (A2165, A2-165, CNCM | 4543, Indica, AHMP-21_2, CNCM | 4574), in cluster 4 (CNCM | 4541, CNCM | 4546, CNCM | 4573, CNCM | 4644, SL3/3, M21/2, and 2789STDY5834970), and in cluster 5 (L2-6, 2789STDY5608869, CNCM | 4575, and AHMP-21).

The clustering model supported by the homology data of *F. prausnitzii* L2-6 (Figure 6) is somewhat different from the above described for *F. prausnitzii* SL3-3. The two models present some similar grouping of strains, i.e., the clusters 0 (dark green colored), 3 (blue colored), and 5 (yellow colored). Moerover, the strains *F. prausnitzii* CNCM I 4540, *F. prausnitzii* CNCM I 4542, *F. prausnitzii* CNCM I 4544, *F. prausnitzii* KLEI1255, *F. prausnitzii* UBA4703, and *F. prausnitzii* UBA1082 are grouped together in both models, namely in cluster 2 (pink colored). However, in the first model, this cluster also contains the strain *F. prausnitzii* CNCM I 4541 whereas in the second model this cluster contains the strain *F. prausnitzii* 60_20.

The strains showing the most different/unique traits (i.e., those more distant from the rest of strains) are the same, i.e., the strains *F. prausnitzii* 60_20, *F. prausnitzii* 58_8, *F. prausnitzii* 61_16, and *F. prausnitzii* 2789STDY5834930. Also interesting, the strains *F. prausnitzii* 61_16 and *F. prausnitzii* 60_20 are together when the *F. prausnitzii* SL3-3 is used as reference (cluster 1, colored in orange), whereas, for the *F. prausnitzii* L2-6, the strain *F. prausnitzii* 61_16 is alone (cluster 1, colored in orange), and the strain *F. prausnitzii* 60_20 belongs to cluster 2. Looking into the *F. prausnitzii* SL3-3 homology data, the strains *F. prausnitzii* 61_16 and *F. prausnitzii* 60_20 show similar high homology (>93%) with a predicted unsaturated glucuronyl hydrolase (FPR_02050), an adenine/guanine phosphoribosyltransferase (FPR_06700) and a thymidine phosphorylase (FPR_21760), and similar low homology (<55%) for a 4-O-beta-D-mannosyl-D-glucose phosphorylase (FPR_17220) and a beta-1,4-mannooligosaccharide/beta-1,4-mannosyl-N-acetylglucosamine phosphorylase (FPR_17240). In turn, in the second analysis, the two strains present quite different glycolytic profiles. For example, the homology scores obtained for a glycogen/starch/alpha-glucan phosphorylase (FP2_13480) and an alpha-glucosidase (FP2_19180) differ in 40%.

See Supplementary Data Sheet S5 for detailed information on this analysis.

### Gene Neighboring

**FIGURE 7:**
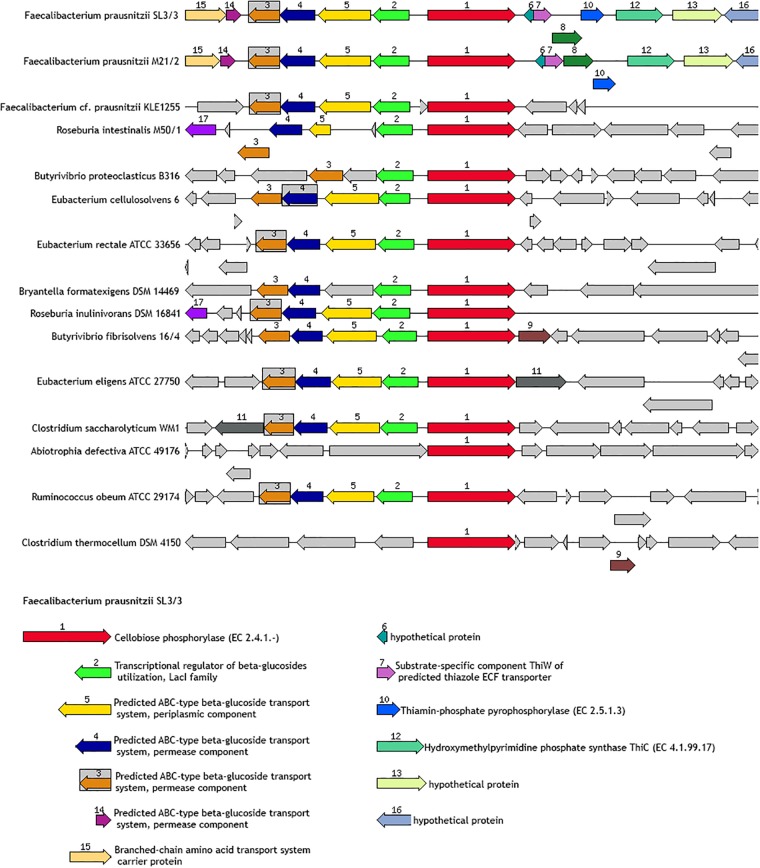
Schematic of the gene neighbor of FPR_05900 (EC.2.4.1.20). This gene encodes a predicted unsatured glucuronyl hydrolase involved in the regulation of bacterial surface properties and related properties.

Genomic neighborhoods can provide an important level of information about the cooperative role of genes in metabolic routes. Notably, functionally related genes are often organized into co-expression functional networks. Therefore, the study of the gene neighborhoods related to certain glycoside hydrolases, either very frequent or very specific in the present collection of genomes, was interesting to gain a better understanding about the genes that participate in the regulation or are functionally related to those genes encoding these activities. In this regard, the SEED genome annotation viewer is a useful tool to investigate how glycoside hydrolase genes are co-localized with other key genes in the *F. prausnitzii* genomes. As an example, a graphical representation of a potential cellobiose utilization cluster is included in Figure 7, showing that genes coding for an ABC transporter, a LacI family transcriptional regulator and a cellobiose phosphorylase (responsible for the phosphorylation of cellobiose and the release of alpha-D-glucose 1-phosphate and D-glucose) seem to be involved in cellobiose uptake. Remarkably, this gene cluster organization is a common feature in other intestinal bacteria (Figure 7). Supplementary Data Sheet S6 presents other examples of gene clusters related to glycoside hydrolase activities in *F. prausnitzii*.

## Discussion

Since *F. prausnitzii* emerged as one of the most promising next-generation probiotics, the research efforts invested in gaining a deeper understanding about the metabolism of this microorganism have proliferated. Till date, and to the best of our knowledge, the glycolytic activities of *F. prausnitzii* had not been portrayed.

Therefore, this work presented an *in silico* approach to the systematic and large-scale study of such metabolic activities in the publicly available genomes. Such approach combined sequence similarity analysis, data clustering and gene neighborhood analysis and took advantage on publicly available functional enzyme and pathway annotations. In total, this study screend the activity of 337 glycoside hydrolases in 27 strains, having two possible strains of reference, i.e., the strains *F. prausnitzii* SL3/3 and *F. prausnitzii* L2-6.

Although information about the carbohydrate preferences of *F. prausnitzii* is scarce, the *in silico* metabolic predictions inferred here are consistent with what we know about the impact of fermentable sugars on the growth promotion and metabolism of *F. prausnitzii.* For instance, the broad representation of glycoside hydrolases potentially involved in the metabolism of galactose-containing oligosaccharides points to a theoretical capability to hydrolyse these kind of substrates. Homologs of the beta-D-galactosidase (FPR_27540; EC.3.2.1 23) and the lacto-N-biose phosphorylase (FPR_27530; EC.2.4.1.211) were present in the majority of strains analyzed. Beta-D-galactosidases release terminal non-reducing β-D-galactose residues in galactosides, such as some prebiotic galactooligosaccharides (Garrido et al., 2013), and lactose-N-biose phosphorylase is involved in the metabolism of lacto-N-biose, the major building block of human milk oligosaccharides (Xiao et al., 2010). In this regard, *in vitro* fermentation experiments and *in vivo* intervention studies highlighted the ability of galactooligosacharides to increase the population of *F. prausnitzii* in the human gut microbiota (Azcarate-Peril et al., 2017; Grimaldi et al., 2017). Also, a recent report shows that there is an increase in bacterial taxa belonging to the species *Faecalibacterium* in the gut microbiota of piglets when fed with key human milk oligosaccharides (Jacobi et al., 2016). This suggests that milk oligosaccharides can contribute to the persistence of *F. prausnitzii* in the intestine of suckling pigs, and therefore, trigger the health promoting effects attributed to this bacterium. In relation to the lacto-N-biose phosphorylase activity, it is also worth mentioning that enzymes involved in the galacto N-biose/lacto-N-biose pathway play a crucial role in the metabolism of mucin from epithelial cells, an important colonization factor in intestinal bacteria (Turroni et al., 2010; Duranti et al., 2015).

Other commonly used prebiotic substrates are inulin-type fructans, which are linear fructans with beta (2←1) fructosyl-fructose linkages, including oligofructose (normally with a chain length of 2–8) and inulin (with a chain length up to 60 moieties). A starting alfa-D-glucose moiety can be present in the backbone but it is not essential (Roberfroid, 2007). Inulin-type fructans have been found to be metabolized by *F. prausnitzii* (Dewulf et al., 2013) and to increase the *Faecalibacterium* population within the human microbiota in intervention studies (Ramirez-Farias et al., 2009; Dewulf et al., 2013; Clarke et al., 2017; Healey et al., 2018). In this regard, the proposed bioinformatics approach identified homologs of alfa and beta glucosidases widely distributed in the 27 genomes of the strains analyzed. These enzymes could be involved in inulin-type fructan degradation (Saulnier et al., 2007; Giraldo et al., 2014). Altogether, the broad presence of the glycoside hydrolase activities discussed above in the *F. prausnitzii* genomes suggests that this species possesses ecological traits that favor its adaptation to the gut environment.

Moreover, a wide representation of strains within the *F. prausnitzii* species. Furthermore, the gene neighboring analysis enabled the description of the genetic context surrounding glycoside hydrolase genes. In present analysis detected glycoside hydrolases potentially involved in the metabolism of maltose (FPR_07280; EC.3.2.1.20) and cellobiose (FPR_05900; EC.2.4.1.20) in the genomes of all the strains analyzed. Maltose and cellobiose are added in culture media for *F. prausnitzii* since these disaccharides specifically promote its growth in laboratory conditions (Martín et al., 2017). Our results support the fact that maltose and cellobiose utilization can be a common metabolic characteristic of *F. prausnitzii*, rather than a strain-dependent feature, and highlight the importance of including this kind of carbon sources for the isolation particular, genes potentially involved in oligosaccharide metabolism are organized in clusters, which are specific to the uptake and degradation of the substrates. This is in accordance with what has been described for other intestinal bacteria (Pokusaeva et al., 2011; Wei et al., 2014).

In summary, although the suitability of our *in silico* approach to infer glycoside hydrolase functional maps of *F. prausnizii* needs to be validated using culturing methods, the results showed in this work are in agreement with the current, limited knowledge on carbohydrate metabolism in this species. After experimental validation, our bioinformatics approach may be reproduced and scaled in order to accommodate the analysis of other strains (or even families and genus), as well as other metabolic activities. This will allow the exploration of novel methodologies to design or obtain targeted prebiotics for *F. prausnitzii* and other strains of interest.

## Author Contributions

BS, FF-R, AM, and AL conceived and designed the study. GB compiled the data and executed all the analyses. All authors drafted the manuscript and read and approved the final version of the manuscript.

## Conflict of Interest Statement

BS and AM are on the scientific board and are co-founders of Microviable Therapeutics SL. The remaining authors declare that the research was conducted in the absence of any commercial or financial relationships that could be construed as a potential conflict of interest.

## Footnotes

1 https://www.genome.jp/kegg/mapper.html

2 http://pubseed.theseed.org/

3 http://rast.nmpdr.org

4 http://rast.nmpdr.org/seedviewer.cgi

## Supplementary Material

TABLE S1 List of the amino acid sequences of the glycoside hydrolases in two fasta files fpa.fasta (corresponds to *F. prausnitzii* SL3/3) and fpr.fasta (corresponds to *F. prausntizii* L2-6).

TABLE S2 Extended analysis of the homology among *F. prausntizii* strains.

TABLE S3 Graphic representation of the clusters and the Davis-Boulding index.

TABLE S4 List of SEED URLs shown for the gene neighbourhood.

DATA SHEET S1 Analysis of *F. prausnitzii* gene orthology.

DATA SHEET S2 Differents examples of gene neighbourhoods related to glycoside hydrolase activities en *F. prausnitzii*.
