# Supplementary material for: In silico Approach for Unveiling the Glycoside Hydrolase Activities in Faecalibacterium prausnitzii Through a Systematic and Integrative Large-Scale Analysis
Source: Front Microbiol. 2019 Apr 4;10:517. doi: 10.3389/fmicb.2019.00517 (PMC6460054; doi:10.3389/fmicb.2019.00517)

FPR_27540 (EC.3.2.1.23). beta-galactosidasa.

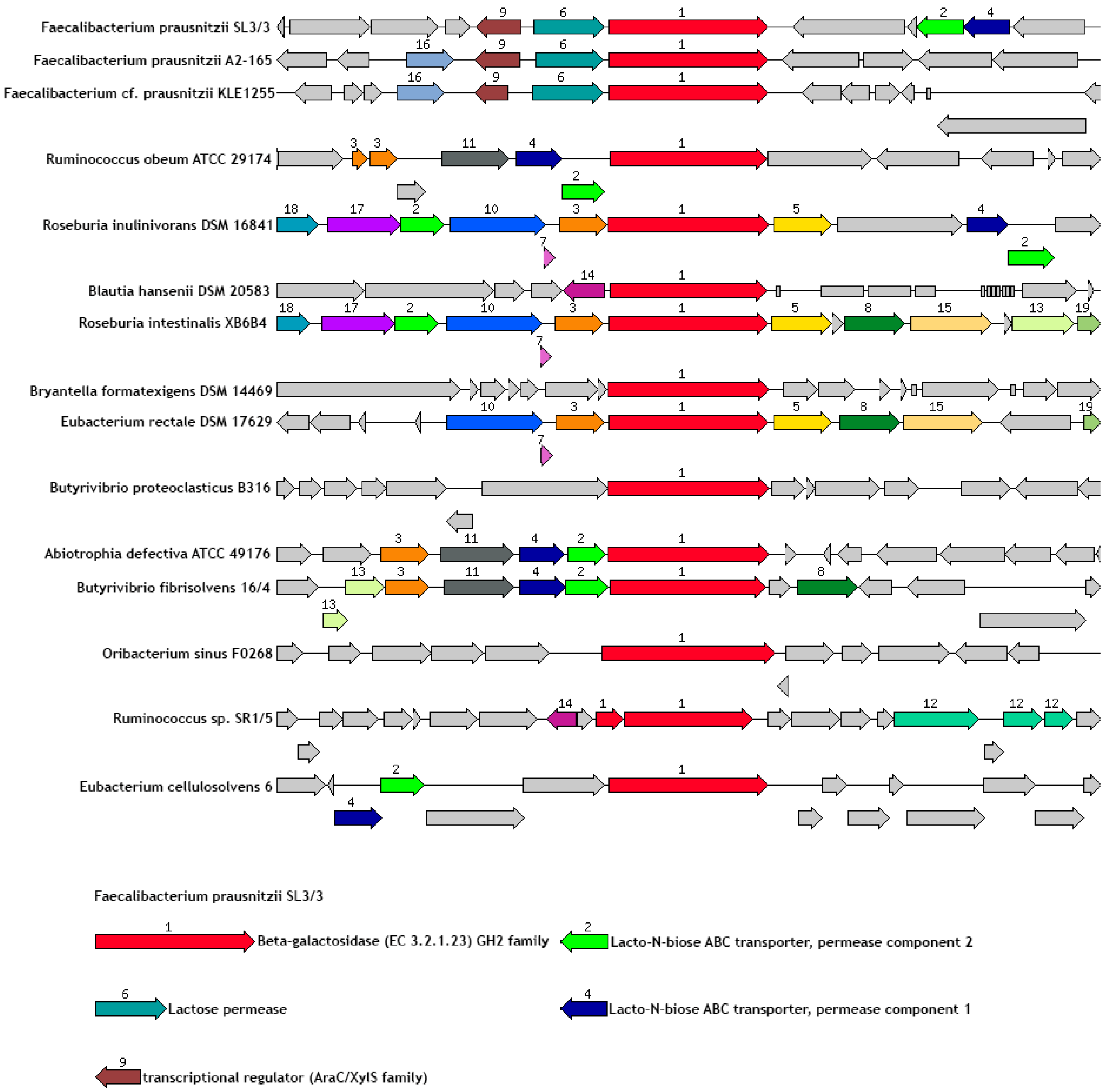

FPR_27530 (EC.2.4.1.211). lacto-N-biosa fosforilasa.

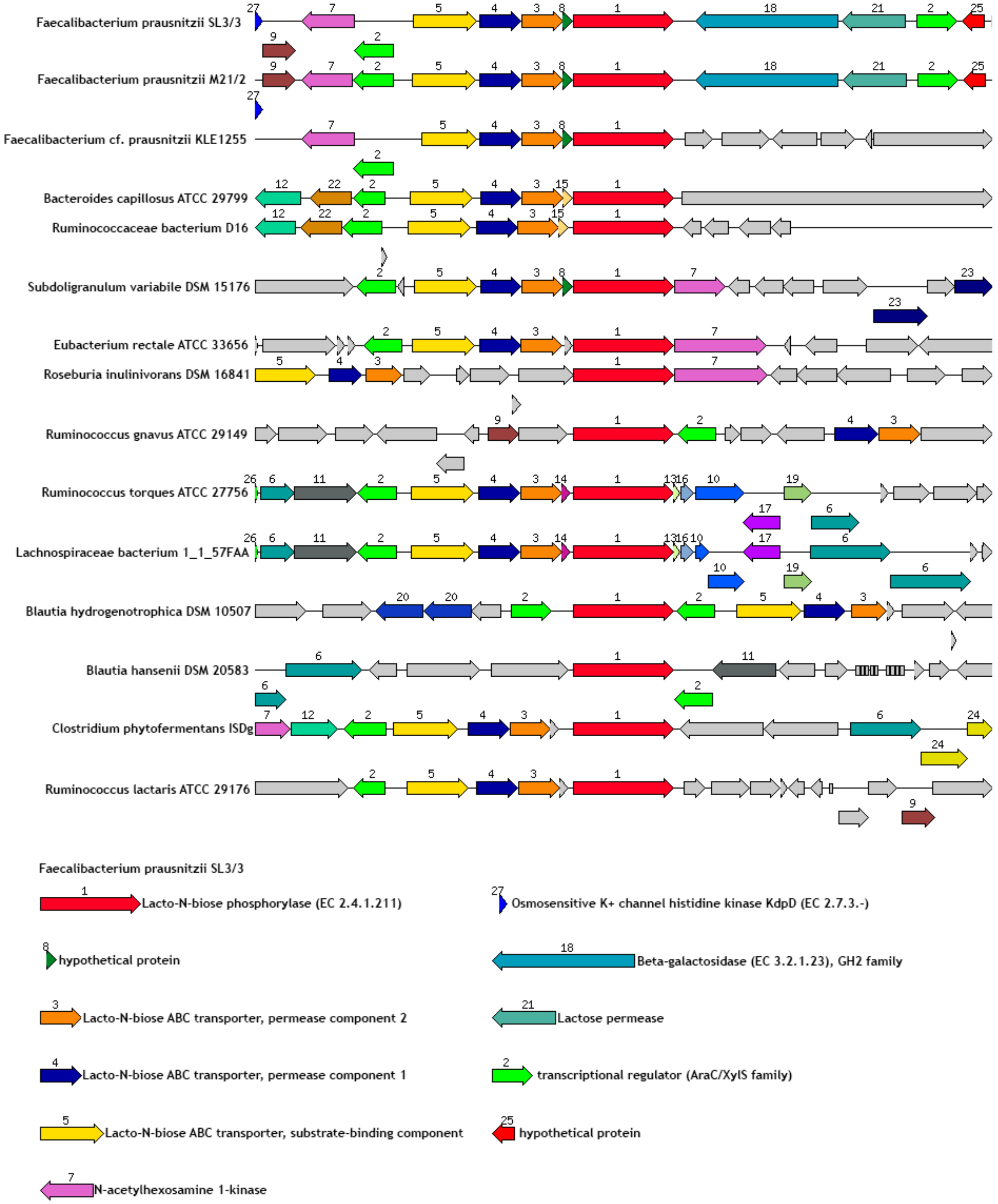

FPR_07280 (EC.3.2.1.20). Metabolismo de maltosa.

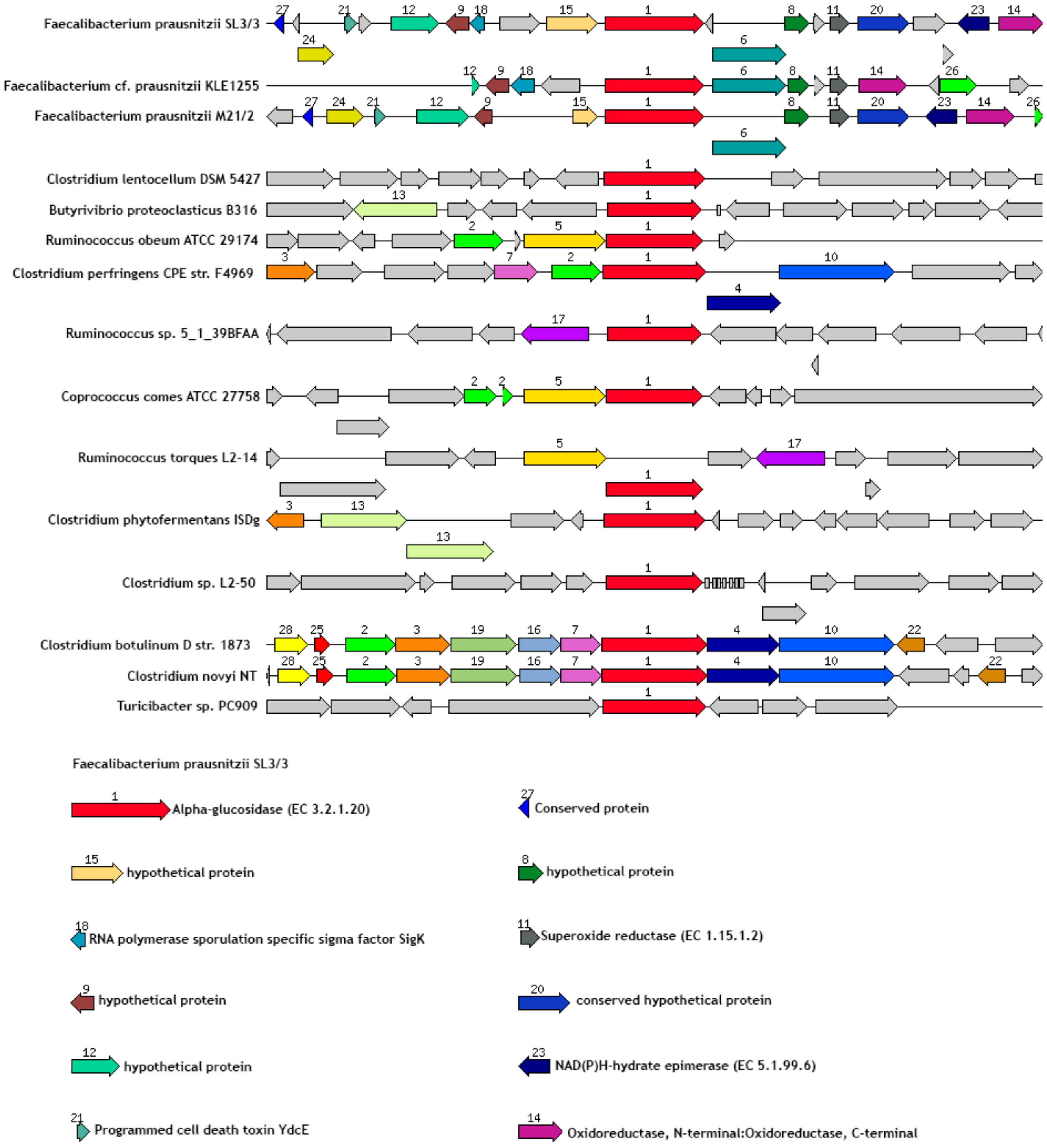

Supplement: DATA SHEET S2 — Differents examples of gene neighbourhoods related to glycoside hydrolase activities en F. prausnitzii. [file Data_Sheet_2.PDF]
